# Women in Surgical Fields; Time to break the Glass Ceiling

**DOI:** 10.12669/pjms.37.5.4583

**Published:** 2021

**Authors:** Tayyaba Gul Malik

**Affiliations:** Prof.Tayyaba Gul Malik, FCPS Ophthalmology Department, Ameer ud din Medical College/Lahore General Hospital, Lahore, Pakistan. Editor, Pakistan Journal of Ophthalmology Email: tayyabam@yahoo.com

Equality or inequality of men and women in different religions, societies, cultures and professions has always been a subject of never-ending debate. From the pre-historic times, women were kept deprived of basic rights. The situation was aggravated when the male dominant societies misinterpreted certain religious teachings in favor of their benefit and used them to exploit female were misinterpreted by the male dominant societies. While Islam discouraged the exploitation of women as a centerpiece of attraction, it made the society realized that women of all ranks and cast deserved to be treated with respect and dignity. Keeping things in these perspective Islam allows females to work. In Holy Quran, it says: “For men there is reward for what they have earned, (and likewise) for women there is reward for what they have earned.”(Al-Quran 4;32)

Right from the beginning of human societies, women have suffered a lot due to the gender discrepancy. The condition exists in its all forms and at all times. Even today in the most modern and civilized era in the history of mankind, gender difference not only exists in cultures and societies but it has remained a hot topic in all walks of life. As the societies started to develop, there was a gradual change in the attitude of men towards women in the professional life. Women started to receive acceptance as nurses because of their gentle nature and caring demeanor. Later they were allowed to get themselves educated but not to acquire higher education. Even then they continued their efforts and proved themselves as good teachers and workers until a time came when they started to support and compete with men in all branches of science with greater preference towards medicine and then surgery. With this development, female surgeons became a topic of discussion at patients’ level, in families and within the surgical departments. Even at the present times, the only cases in which female surgeons are sought for, are breast or cervical cancer. Otherwise, there is a general concept that male surgeons are better than females.

If we look back into the history, we find at one end of the spectrum that women surgeons existed in 3500 BC as evident from the remains recovered from the grave of Queen Shubad of Ur. There were metal surgical instruments, which were buried with her to continue her surgical practice in life after death.^1^ At the other end, we have a very inspiring story of Dr. James Barry who fought the gender bias in an extraordinary way. Dr. James was the person who performed one of the first successful cesarean sections in the history of medical science. After graduation, he joined army as a surgeon and served for his whole life. After his death, it was found that Dr. James was a female and had evidence of previous pregnancy as well. She was Dr. Miranda Stewart.^2^ Another example is that of Dr. Elizabeth Blackwell (1821–1910) who was rejected from more than 20 medical institutions because of her gender. At last, she was admitted in a medical school and got gold medal from her college but could not practice and ultimately had to serve as an obstetrical nurse.^3^ These are just a few examples. While the females were being kept back from surgical fields, the female surgeons did not surrender. They continued their efforts to get themselves recognized. One of the many examples is Tortula who in the eleventh century, wrote practice of gynecology and midwifery, which was read for a long time afterwards.^4^ Even with their contributions in this field, women were banned from practicing surgery for a very long time. During the 14^th^ and 15^th^ century, women had to take permission from jury to practice. These permissions were conditional, only to carry out the process of their late husbands.^5^ Similar was the situation in other parts of the world.

Now situation has been changing gradually throughout the world as the number of female doctors entering the medical schools has been considerably improved over the last many decades but there is no proportionate increase in the number of female surgeons. It is only in the field of Obstetrics and Gynecology that women outnumber males.

If we take the example of Pakistan, according to the Pakistan Medical and Dental Council (PMDC), more than 70% of medical students are women. Out of this huge percentage, a very small proportion enters the surgical specialties. A recent survey done in Karachi, showed that 72% of female surgeons felt that there were cultural barriers for their being adopting surgery as a career. These barriers were related with their families and particularly having children. Eighty-six percent of female doctors were told that surgery was a field suited for males and females can only become good obstetricians, gynecologists and pediatricians. The toxicity increases when it is also assumed that men are more intelligent and females do not have the capability of becoming good surgeons. Their skills are also questioned without being tested creating a negative atmosphere for women.^6^ According to PMDC, during 2006-2008, out of 1,331 registrations in surgical specialties, there were only 225 women. In 2017, there were 344 men and 70 women and in 2018, 379 men and only 82 women.^7^

There has been a general misconception that surgery is not a welcoming field for the females and this holds true for all parts of the world. As worldwide data shows that only one third of surgeons make up female surgeons.^8^ Women who are interested in surgery are first of all faced by the family resistance that they are not welcomed in this field, secondly they are shown a list of responsibilities they have to consider, later if they manage to join surgical field, they are subjected to gender discrimination in training programs and in promotions.

The situation is not different in high-income countries. The percentage of female surgeons has increased to only 27% by 2017. In Germany, the percentage was 21.1 in 2018.^9^ According to association of American medical colleges AAMC 2017 data, women were less common in surgical specialties. It was also noted that as the female surgeons moved up the ladder of promotions, gender discrimination increased. Cause is again the mindset that a female being a less capable human cannot take up higher ranks and it is still unacceptable to the males. At higher positions, females less commonly reach the chair positions. In 2016, only 20 females were head of Departments of Surgery in the United States.^10^ In the UK only 11 % were heading the surgical departments in 2016.^10^

Gender pay gap in the surgical specialties is another dilemma faced by female surgeons of the west. According to CNN report, females were paid less as compared to their male counterparts even though at an average they spent two minutes more on every patient.^11^ Situation is comparatively better in Pakistan regarding pay. Government has set rules for paid maternity leaves of three months, which is a very good incentive.

Apart from gender bias, there are other factors contributing to lesser number of females in surgery; work-life balance, family and children issues, pregnancy etc. There are many surgical departments in which female residents are discouraged on marriage and family grounds. To overcome this, Pakistan government has women quota in different fields including medicine and surgery, which has again become a source of discontent and resentment in the male colleagues.

When all this negativity is being talked about, people forget the shining examples of females who despite hardships in their lives had done great job in surgical fields. One such name is Isabel Hayes Chapin Barrows who was the first American ophthalmologist. She had a lot of firsts in her lifetime including being the first woman to be admitted to the University of Vienna in Austria to study ophthalmology. She was an editor, reporter, prison reformer, ambassador and a mother of two children.^12^ Another example is Deborah Pavan Langston who was the first female resident in ophthalmic field and later she was recognized as an authority in ocular viral diseases.

There are few encouraging reports for women as well. According to a recent study, performance of female surgeons was better in early post-operative outcomes. There was lesser 30-day mortality, lesser complications and length of stay at hospital in patients treated by female surgeons.^13^ It was also seen that in communication skills, medical knowledge, technical skills, clinical judgement and professionalism, women had shown equal competence.

In a country like Pakistan where there are enormous financial issues, keeping women out of surgical fields may add up the economical loss or burden country has to face by avoiding entry of women in surgery. There are social issues as well which include, majority of rural women feeling uncomfortable to be operated by male surgeons.

There is a long list of challenges women surgeons have to face. Due to the complexity of these problems, the pathway to find a plausible solution becomes more complicated. If the problems are clearly defined, only then the solution can be sought. Mind set has to be changed at every level to break the glass ceiling.
